# Vulvar Lipoma: A Case Report

**DOI:** 10.1055/s-0038-1670642

**Published:** 2018-10

**Authors:** Ahmed Reda, Ihab Gomaa

**Affiliations:** 1 Department of Obstetrics and Gynecology, Ain Shams University, Cairo, Egypt

**Keywords:** benign tumor, lipoma, mesenchymal tissue, soft tissue, vulva, vulvar lipoma

## Abstract

The present study is a case report of vulvar lipoma. The vulva is a rare site for the development of lipomas, and the aim of the study is to determine if the current imaging modalities can diagnose lipomas correctly. A 43-year-old patient presented with a painless, slowly progressive, oval, mobile and non-tender right vulvar mass compressing the vagina and totally covering the introitus. Both the ultrasonography and magnetic resonance imaging (MRI) exams suggested the diagnosis of lipoma. Surgical excision was performed, and the histopathological examination of the mass confirmed a lipoma.

## Introduction

Lipoma is a common benign tumor of soft tissue, and its diagnosis is confirmed by the histopathological description of a well-circumscribed collection of mature adipose tissue.^1^ The etiology of lipoma is still to be elucidated, but it has been reported that trauma^2^ and gene rearrangement^3^ may play a role in its development.

Typically found in subcutaneous fat, lipoma is frequently observed in the upper back, the neck, and the proximal upper and lower extremities.^3^ The vulva is a rare site for the development of lipomas, with few cases reported in the literature.^4^ ^5^ ^6^ ^7^ ^8^ ^9^ The differential diagnosis of vulvar lipoma includes Bartholin gland cyst and abscess, inguinal hernia, as well as several benign and malignant neoplastic conditions.^10^

## Case Description

A 43–year-old woman presented to our clinic complaining of a vulvar mass that lately had been causing her discomfort during walking and sitting. The mass was painless, with an insidious onset and slowly progressive course over 4 years. The woman is a house wife, p5, with no past history of clinical importance. There was no past personal or family history of a similar condition.

**Fig. 1:**
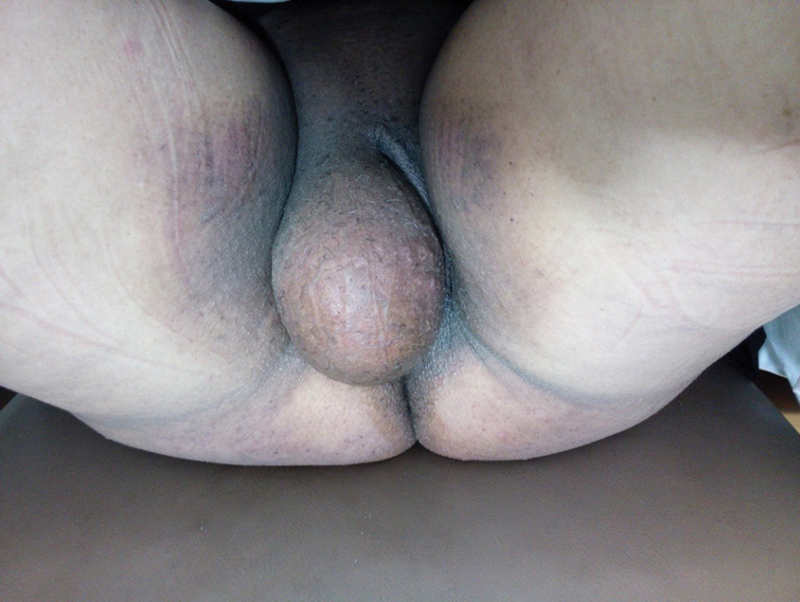
Vulvar swelling occupying the whole right labia majora and covering the introitus.

Upon examination, there was a single oval mass involving the whole right labia majora and covering the introitus (Fig. 1). The mass measured ∼ 15 cm in diameter, and was soft, mobile, non-tender, non-reducible, and gave no impulse on cough. The overlying skin was mobile, but stretched over this large mass. The mass was compressing the vagina, shifting it to the opposite side, but it was not fixed to the vagina. There was no palpable inguinal lymph node.

**Fig. 2:**
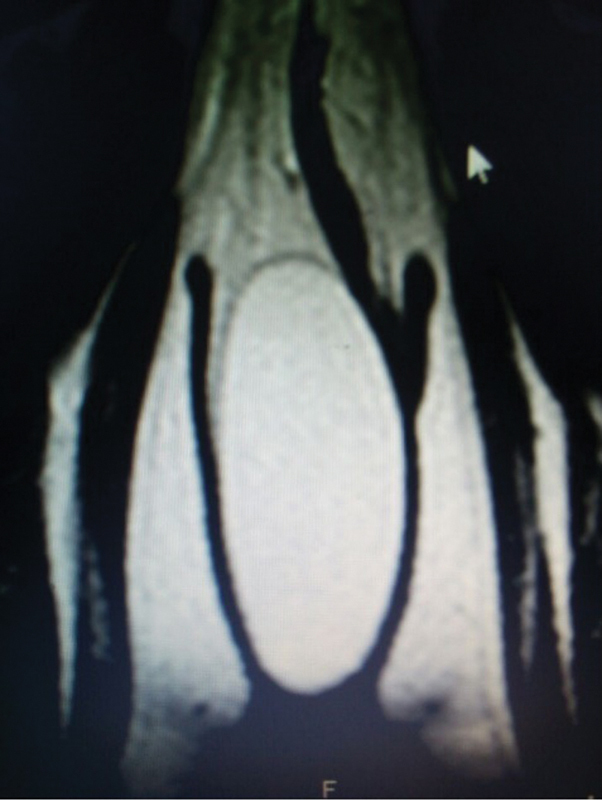
Magnetic resonance imaging scan showing oval soft tissue mass with well-defined capsule.

An ultrasonographic examination showed a homogenous echo texture with no cystic changes or vascularity surrounded by an echogenic capsule with minimal vascularity. A magnetic resonance imaging (MRI) scan revealed an oval soft tissue mass with a well-defined capsule showing a homogenous high signal with suppression of a signal similar to subcutaneous fat, and showing no diffusion restriction or postcontrast enhancement (Fig. 2).

**Fig. 3:**
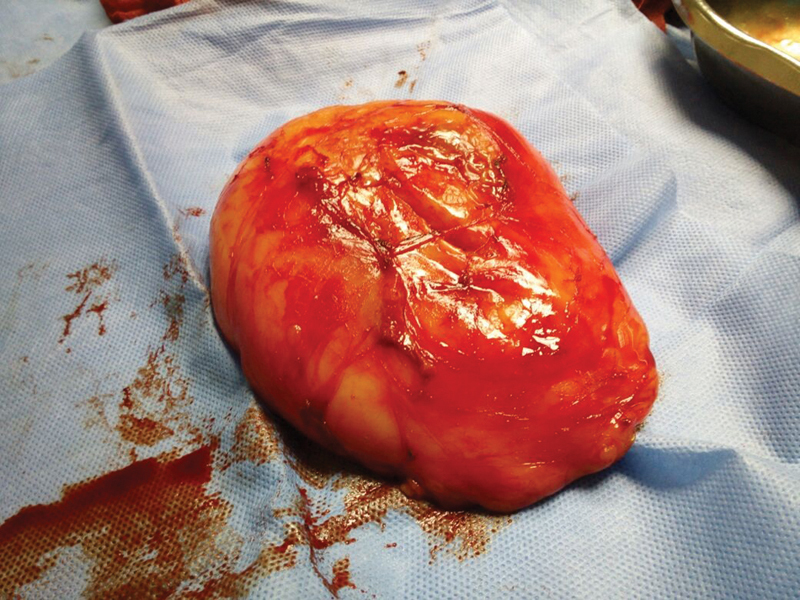
Vulvar lipoma after surgical excision.

The patient underwent surgical excision; an elliptical incision on the skin was performed to aid dissection and to remove redundant tissue for better cosmetic closure. The mass was easily separated from the surrounding tissue, and was removed completely from its capsule (Fig. 3). The histopathological examination revealed a collection of mature adipose tissue, confirming the diagnosis of lipoma.

## Discussion

Mesenchymal tissue, also referred to as soft tissue or connective tissue, is a derivative of the mesoderm. It comprises a variety of cell types, including fibroblasts and adipocytes. The most frequent benign mesenchymal tumor is lipoma, which is typically observed in subcutaneous fat, but may develop in almost all body organs, including the vulva, which is a rare site for lipoma development.

The etiology and pathogenesis of lipomas remain undetermined. Trauma, obesity and gene rearrangement are risk factors for the development of lipomas.^2^ ^3^ In the current report, we could not link lipoma to any risk factor. Similarly, previous case reports of vulvar lipoma did not find associated risk factors. Only one study^7^ suggested trauma might be linked to their patient, a 17-year-old Tae Kwon Do practitioner.

Lipoma commonly develops between the fourth and sixth decades of life, but it can be found in all age groups. Like other lipomas, vulvar lipoma has a similar pattern, being reported in all age groups,^4^ ^5^ ^6^ ^7^ ^8^ ^9^ including adolescents^7^ ^8^ and infants.^9^

The diagnosis of lipoma is usually made through a clinical examination. It may present as a painless mass that has a slowly progressive course and is soft, mobile and not adherent to the overlying skin. In the current case, the clinical examination was suggestive of lipoma, but due to the rarity of the condition, we preferred not to rely on clinical findings alone, and decided to subject the patient to further imaging investigations.

The ultrasound examination of a case report of vulvar lipoma showed that the appearance of an encapsulated homogenous echogenic mass is a diagnostic criterion for lipoma.^11^ An earlier study on ultrasonography of superficial lipomas revealed that the majority of the cases had homogenous echotexture and were well-defined masses.^12^ The MRI examination of lipomas usually shows high signal in weighted images, and serves as the best imaging modality for the assessment of soft tissue.^13^ In the present study, both imaging modalities were excellent in diagnosing vulvar lipoma.

Lipomas can be managed conservatively, especially if they are small in size and asymptomatic, for, if they grow large, they may cause discomfort and disfigurement, and may result in psychological and social problems. Surgical excision, liposuction, laser, ultrasound and injection of pharmaceutical agents are management options for the treatment of lipomas.^14^ Surgical excision is the treatment of choice for lipomas, with complete removal of the capsule to prevent recurrence. In the present case, we chose surgical excision because the mass was symptomatic, and to confirm the diagnosis of lipoma, since the vulva is a rare site for the development of this tumor.

## Conclusion

The vulva is a rare site for the development of lipomas. Ultrasonography and MRI are the preferred diagnostic modalities for lipoma. In symptomatic cases, surgical excision is the treatment of choice.
